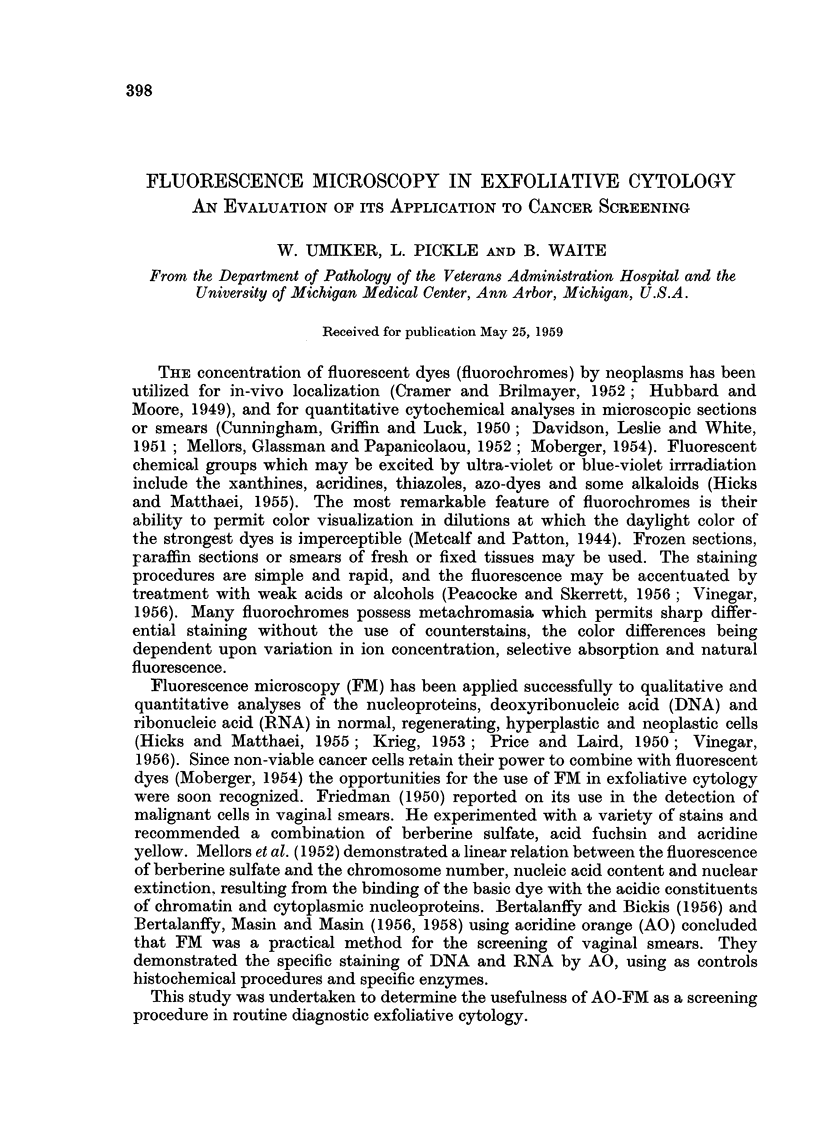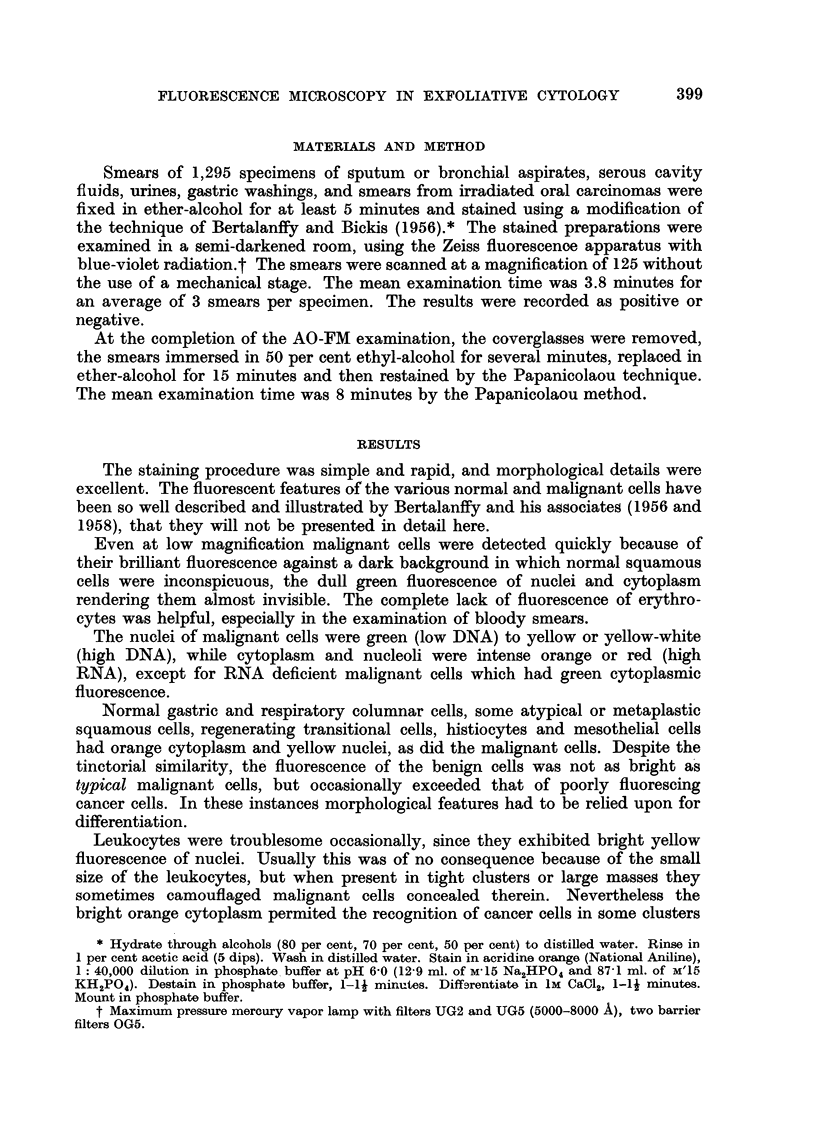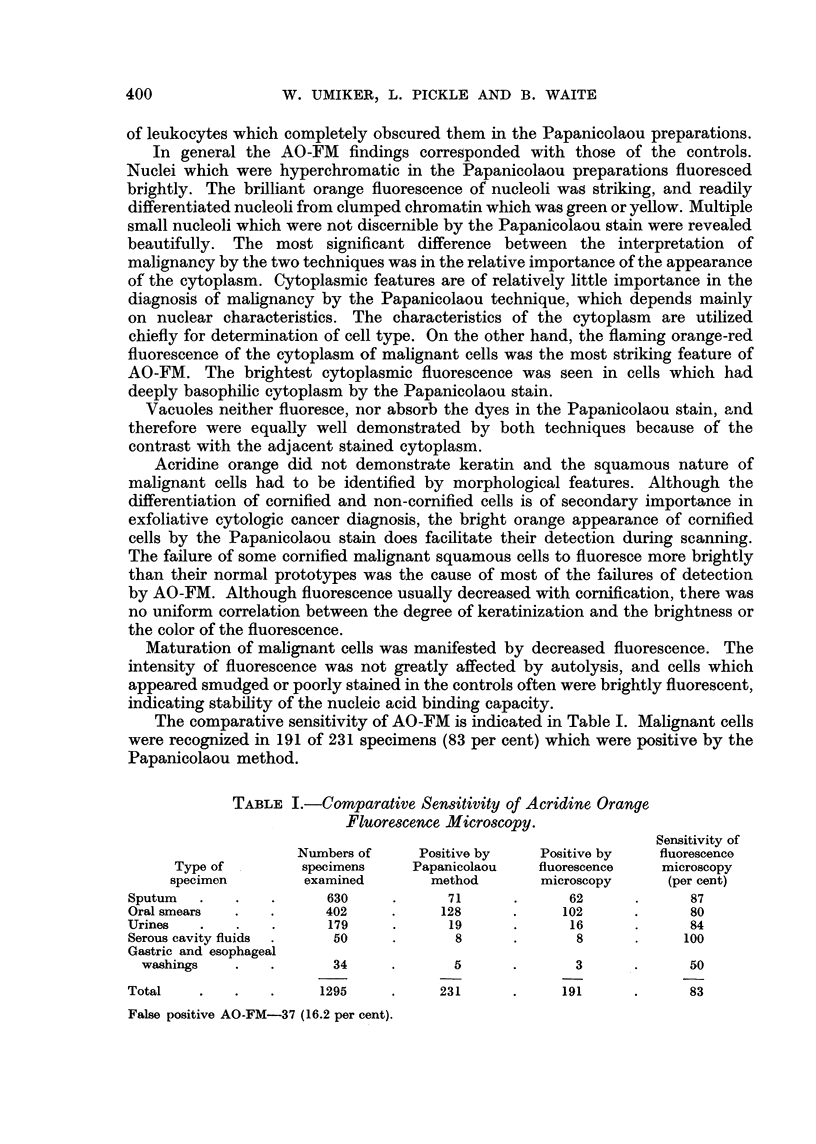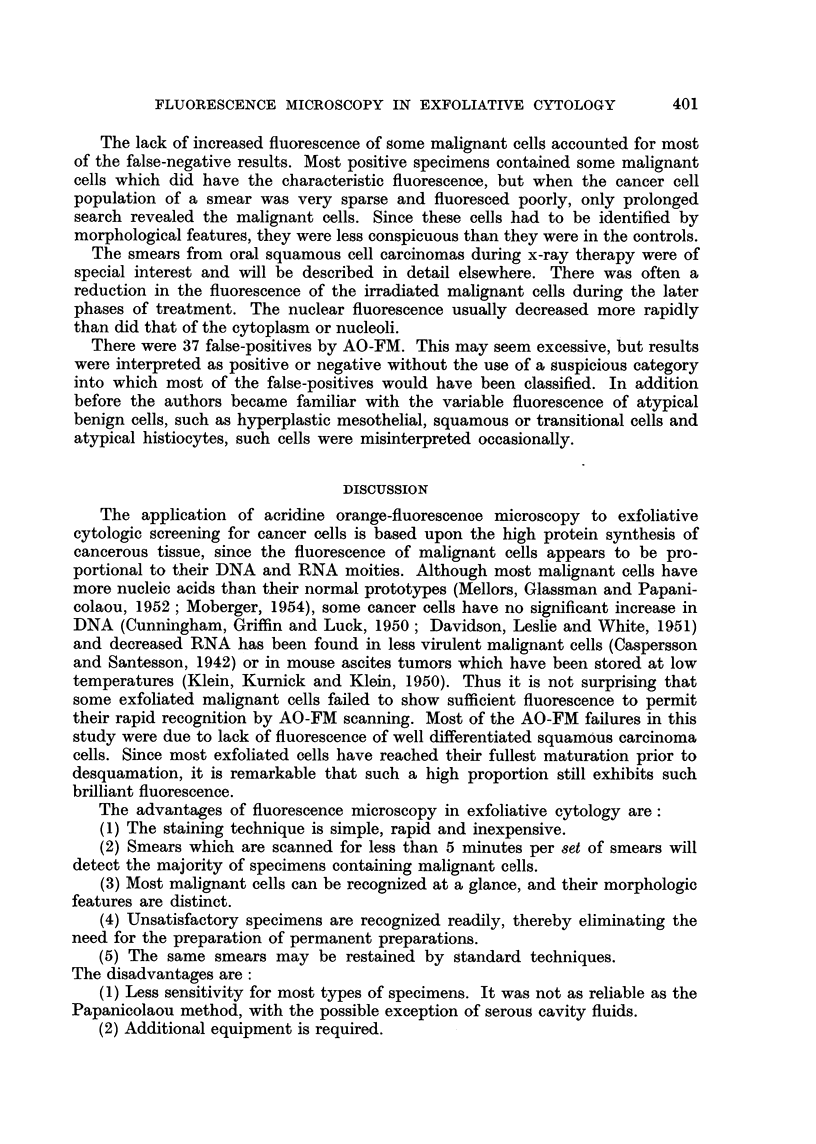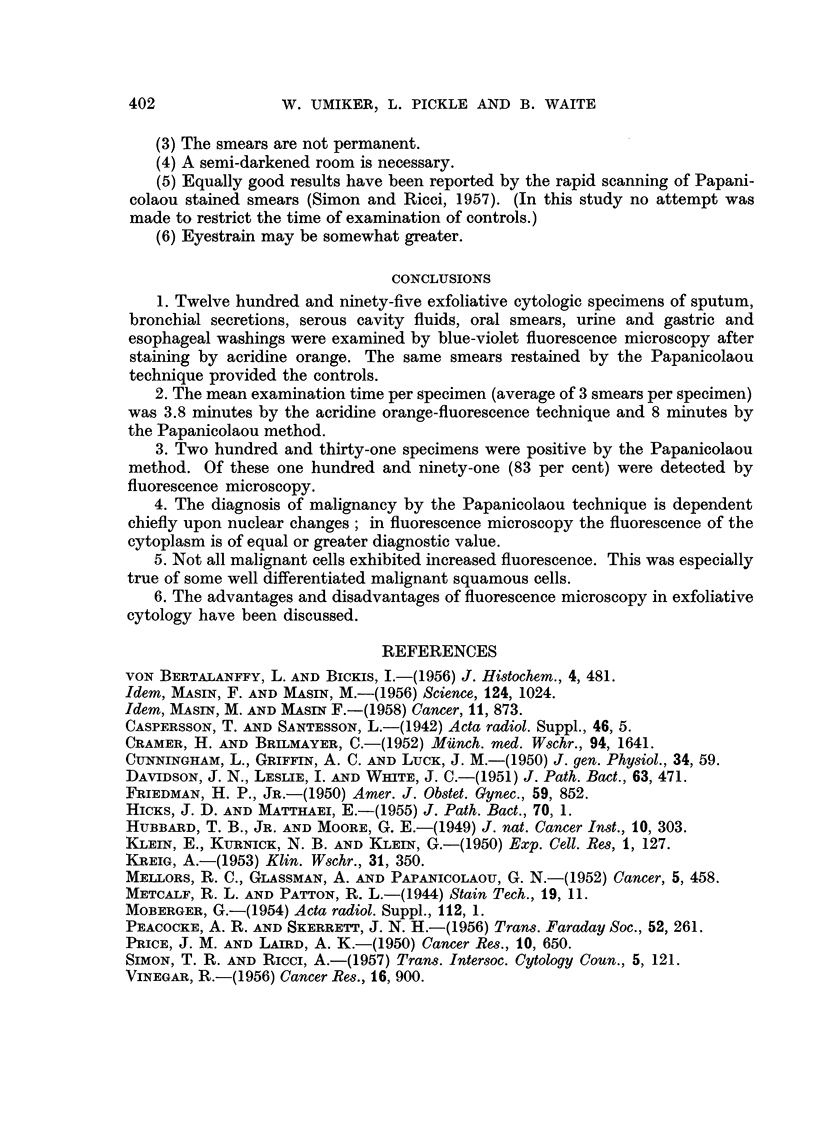# Fluorescence Microscopy in Exfoliative Cytology

**DOI:** 10.1038/bjc.1959.45

**Published:** 1959-09

**Authors:** W. Umiker, L. Pickle, B. Waite


### FLUORESCENCE MICROSCOPY IN EXFOLIATIVE CYTOLOGY

### AN EVALUATION OF ITS APPLICATION TO CANCER SCREENING

W. UMIKER, L. PICKLE AND B. WAITE

*From the Department of Pathology of the Veterans Administration Hospital and the University of Michigan Medical Center, Ann Arbor, Michigan, U.S.A.*

Received for publication May 25, 1959

THE concentration of fluorescent dyes (fluorochromes) by neoplasms has been utilized for in-vivo localization (Cramer and Brilmayer, 1952 ; Hubbard and Moore, 1949), and for quantitative cytochemical analyses in microscopic sections or smears (Cunningham, Griffin and Luck, 1950 ; Davidson, Leslie and White, 1951 ; Mellors, Glassman and Papanicolaou, 1952 ; Moberger, 1954). Fluorescent chemical groups which may be excited by ultra-violet or blue-violet irradiation include the xanthines, acridines, thiazoles, azo-dyes and some alkaloids (Hicks and Matthaei, 1955). The most remarkable feature of fluorochromes is their ability to permit color visualization in dilutions at which the daylight color of the strongest dyes is imperceptible (Metcalf and Patton, 1944). Frozen sections, paraffin sections or smears of fresh or fixed tissues may be used. The staining procedures are simple and rapid, and the fluorescence may be accentuated by treatment with weak acids or alcohols (Peacocke and Skerrett, 1956 ; Vinegar, 1956). Many fluorochromes possess metachromasia which permits sharp differential staining without the use of counterstains, the color differences being dependent upon variation in ion concentration, selective absorption and natural fluorescence.

Fluorescence microscopy (FM) has been applied successfully to qualitative and quantitative analyses of the nucleoproteins, deoxyribonucleic acid (DNA) and ribonucleic acid (RNA) in normal, regenerating, hyperplastic and neoplastic cells (Hicks and Matthaei, 1955 ; Krieg, 1953 ; Price and Laird, 1950 ; Vinegar, 1956). Since non-viable cancer cells retain their power to combine with fluorescent dyes (Moberger, 1954) the opportunities for the use of FM in exfoliative cytology were soon recognized. Friedman (1950) reported on its use in the detection of malignant cells in vaginal smears. He experimented with a variety of stains and recommended a combination of berberine sulfate, acid fuchsin and acridine yellow. Mellors *et al.* (1952) demonstrated a linear relation between the fluorescence of berberine sulfate and the chromosome number, nucleic acid content and nuclear extinction, resulting from the binding of the basic dye with the acidic constituents of chromatin and cytoplasmic nucleoproteins. Bertalanffy and Bickis (1956) and Bertalanffy, Masin and Masin (1956, 1958) using acridine orange (AO) concluded that FM was a practical method for the screening of vaginal smears. They demonstrated the specific staining of DNA and RNA by AO, using as controls histochemical procedures and specific enzymes.

This study was undertaken to determine the usefulness of AO-FM as a screening procedure in routine diagnostic exfoliative cytology.

### MATERIALS AND METHOD

Smears of 1,295 specimens of sputum or bronchial aspirates, serous cavity fluids, urines, gastric washings, and smears from irradiated oral carcinomas were fixed in ether-alcohol for at least 5 minutes and stained using a modification of the technique of Bertalanffy and Bickis (1956).* The stained preparations were examined in a semi-darkened room, using the Zeiss fluorescence apparatus with blue-violet radiation.† The smears were scanned at a magnification of 125 without the use of a mechanical stage. The mean examination time was 3.8 minutes for an average of 3 smears per specimen. The results were recorded as positive or negative.

At the completion of the AO-FM examination, the coverglasses were removed, the smears immersed in 50 per cent ethyl-alcohol for several minutes, replaced in ether-alcohol for 15 minutes and then restained by the Papanicolaou technique. The mean examination time was 8 minutes by the Papanicolaou method.

### RESULTS

The staining procedure was simple and rapid, and morphological details were excellent. The fluorescent features of the various normal and malignant cells have been so well described and illustrated by Bertalanffy and his associates (1956 and 1958), that they will not be presented in detail here.

Even at low magnification malignant cells were detected quickly because of their brilliant fluorescence against a dark background in which normal squamous cells were inconspicuous, the dull green fluorescence of nuclei and cytoplasm rendering them almost invisible. The complete lack of fluorescence of erythrocytes was helpful, especially in the examination of bloody smears.

The nuclei of malignant cells were green (low DNA) to yellow or yellow-white (high DNA), while cytoplasm and nucleoli were intense orange or red (high RNA), except for RNA deficient malignant cells which had green cytoplasmic fluorescence.

Normal gastric and respiratory columnar cells, some atypical or metaplastic squamous cells, regenerating transitional cells, histiocytes and mesothelial cells had orange cytoplasm and yellow nuclei, as did the malignant cells. Despite the tinctorial similarity, the fluorescence of the benign cells was not as bright as *typical* malignant cells, but occasionally exceeded that of poorly fluorescing cancer cells. In these instances morphological features had to be relied upon for differentiation.

Leukocytes were troublesome occasionally, since they exhibited bright yellow fluorescence of nuclei. Usually this was of no consequence because of the small size of the leukocytes, but when present in tight clusters or large masses they sometimes camouflaged malignant cells concealed therein. Nevertheless the bright orange cytoplasm permited the recognition of cancer cells in some clusters

* Hydrate through alcohols (80 per cent, 70 per cent, 50 per cent) to distilled water. Rinse in 1 per cent acetic acid (5 dips). Wash in distilled water. Stain in acridine orange (National Aniline), 1 : 40,000 dilution in phosphate buffer at pH 6·0 (12·9 ml. of M·15 $Na_2HPO_4$ and 87·1 ml. of M′15 $KH_2PO_4$). Destain in phosphate buffer, 1–1½ minutes. Differentiate in 1M $CaCl_2$, 1–1½ minutes. Mount in phosphate buffer.

† Maximum pressure mercury vapor lamp with filters UG2 and UG5 (5000–8000 Å), two barrier filters OG5.

of leukocytes which completely obscured them in the Papanicolaou preparations.

In general the AO-FM findings corresponded with those of the controls. Nuclei which were hyperchromatic in the Papanicolaou preparations fluoresced brightly. The brilliant orange fluorescence of nucleoli was striking, and readily differentiated nucleoli from clumped chromatin which was green or yellow. Multiple small nucleoli which were not discernible by the Papanicolaou stain were revealed beautifully. The most significant difference between the interpretation of malignancy by the two techniques was in the relative importance of the appearance of the cytoplasm. Cytoplasmic features are of relatively little importance in the diagnosis of malignancy by the Papanicolaou technique, which depends mainly on nuclear characteristics. The characteristics of the cytoplasm are utilized chiefly for determination of cell type. On the other hand, the flaming orange-red fluorescence of the cytoplasm of malignant cells was the most striking feature of AO-FM. The brightest cytoplasmic fluorescence was seen in cells which had deeply basophilic cytoplasm by the Papanicolaou stain.

Vacuoles neither fluoresce, nor absorb the dyes in the Papanicolaou stain, and therefore were equally well demonstrated by both techniques because of the contrast with the adjacent stained cytoplasm.

Acridine orange did not demonstrate keratin and the squamous nature of malignant cells had to be identified by morphological features. Although the differentiation of cornified and non-cornified cells is of secondary importance in exfoliative cytologic cancer diagnosis, the bright orange appearance of cornified cells by the Papanicolaou stain does facilitate their detection during scanning. The failure of some cornified malignant squamous cells to fluoresce more brightly than their normal prototypes was the cause of most of the failures of detection by AO-FM. Although fluorescence usually decreased with cornification, there was no uniform correlation between the degree of keratinization and the brightness or the color of the fluorescence.

Maturation of malignant cells was manifested by decreased fluorescence. The intensity of fluorescence was not greatly affected by autolysis, and cells which appeared smudged or poorly stained in the controls often were brightly fluorescent, indicating stability of the nucleic acid binding capacity.

The comparative sensitivity of AO-FM is indicated in Table I. Malignant cells were recognized in 191 of 231 specimens (83 per cent) which were positive by the Papanicolaou method.

TABLE I.—*Comparative Sensitivity of Acridine Orange Fluorescence Microscopy.*

| Type of specimen | Numbers of specimens examined | Positive by Papanicolaou method | Positive by fluorescence microscopy | Sensitivity of fluorescence microscopy (per cent) |
|---|---|---|---|---|
| Sputum | 630 | 71 | 62 | 87 |
| Oral smears | 402 | 128 | 102 | 80 |
| Urines | 179 | 19 | 16 | 84 |
| Serous cavity fluids | 50 | 8 | 8 | 100 |
| Gastric and esophageal washings | 34 | 5 | 3 | 50 |
| Total | 1295 | 231 | 191 | 83 |

False positive AO-FM—37 (16.2 per cent).

The lack of increased fluorescence of some malignant cells accounted for most of the false-negative results. Most positive specimens contained some malignant cells which did have the characteristic fluorescence, but when the cancer cell population of a smear was very sparse and fluoresced poorly, only prolonged search revealed the malignant cells. Since these cells had to be identified by morphological features, they were less conspicuous than they were in the controls.

The smears from oral squamous cell carcinomas during x-ray therapy were of special interest and will be described in detail elsewhere. There was often a reduction in the fluorescence of the irradiated malignant cells during the later phases of treatment. The nuclear fluorescence usually decreased more rapidly than did that of the cytoplasm or nucleoli.

There were 37 false-positives by AO-FM. This may seem excessive, but results were interpreted as positive or negative without the use of a suspicious category into which most of the false-positives would have been classified. In addition before the authors became familiar with the variable fluorescence of atypical benign cells, such as hyperplastic mesothelial, squamous or transitional cells and atypical histiocytes, such cells were misinterpreted occasionally.

### DISCUSSION

The application of acridine orange-fluorescence microscopy to exfoliative cytologic screening for cancer cells is based upon the high protein synthesis of cancerous tissue, since the fluorescence of malignant cells appears to be proportional to their DNA and RNA moities. Although most malignant cells have more nucleic acids than their normal prototypes (Mellors, Glassman and Papanicolaou, 1952 ; Moberger, 1954), some cancer cells have no significant increase in DNA (Cunningham, Griffin and Luck, 1950 ; Davidson, Leslie and White, 1951) and decreased RNA has been found in less virulent malignant cells (Caspersson and Santesson, 1942) or in mouse ascites tumors which have been stored at low temperatures (Klein, Kurnick and Klein, 1950). Thus it is not surprising that some exfoliated malignant cells failed to show sufficient fluorescence to permit their rapid recognition by AO-FM scanning. Most of the AO-FM failures in this study were due to lack of fluorescence of well differentiated squamous carcinoma cells. Since most exfoliated cells have reached their fullest maturation prior to desquamation, it is remarkable that such a high proportion still exhibits such brilliant fluorescence.

The advantages of fluorescence microscopy in exfoliative cytology are :

(1) The staining technique is simple, rapid and inexpensive.

(2) Smears which are scanned for less than 5 minutes per *set* of smears will detect the majority of specimens containing malignant cells.

(3) Most malignant cells can be recognized at a glance, and their morphologic features are distinct.

(4) Unsatisfactory specimens are recognized readily, thereby eliminating the need for the preparation of permanent preparations.

(5) The same smears may be restained by standard techniques.

The disadvantages are :

(1) Less sensitivity for most types of specimens. It was not as reliable as the Papanicolaou method, with the possible exception of serous cavity fluids.

(2) Additional equipment is required.

(3) The smears are not permanent.

(4) A semi-darkened room is necessary.

(5) Equally good results have been reported by the rapid scanning of Papanicolaou stained smears (Simon and Ricci, 1957). (In this study no attempt was made to restrict the time of examination of controls.)

(6) Eyestrain may be somewhat greater.

### CONCLUSIONS

1. Twelve hundred and ninety-five exfoliative cytologic specimens of sputum, bronchial secretions, serous cavity fluids, oral smears, urine and gastric and esophageal washings were examined by blue-violet fluorescence microscopy after staining by acridine orange. The same smears restained by the Papanicolaou technique provided the controls.
2. The mean examination time per specimen (average of 3 smears per specimen) was 3.8 minutes by the acridine orange-fluorescence technique and 8 minutes by the Papanicolaou method.
3. Two hundred and thirty-one specimens were positive by the Papanicolaou method. Of these one hundred and ninety-one (83 per cent) were detected by fluorescence microscopy.
4. The diagnosis of malignancy by the Papanicolaou technique is dependent chiefly upon nuclear changes ; in fluorescence microscopy the fluorescence of the cytoplasm is of equal or greater diagnostic value.
5. Not all malignant cells exhibited increased fluorescence. This was especially true of some well differentiated malignant squamous cells.
6. The advantages and disadvantages of fluorescence microscopy in exfoliative cytology have been discussed.

### REFERENCES

VON BERTALANFFY, L. AND BICKIS, I.—(1956) *J. Histochem.*, **4**, 481.
*Idem*, MASIN, F. AND MASIN, M.—(1956) *Science*, **124**, 1024.
*Idem*, MASIN, M. AND MASIN F.—(1958) *Cancer*, **11**, 873.
CASPERSSON, T. AND SANTESSON, L.—(1942) *Acta radiol.* Suppl., **46**, 5.
CRAMER, H. AND BRILMAYER, C.—(1952) *Münch. med. Wschr.*, **94**, 1641.
CUNNINGHAM, L., GRIFFIN, A. C. AND LUCK, J. M.—(1950) *J. gen. Physiol.*, **34**, 59.
DAVIDSON, J. N., LESLIE, I. AND WHITE, J. C.—(1951) *J. Path. Bact.*, **63**, 471.
FRIEDMAN, H. P., JR.—(1950) *Amer. J. Obstet. Gynec.*, **59**, 852.
HICKS, J. D. AND MATTHAEI, E.—(1955) *J. Path. Bact.*, **70**, 1.
HUBBARD, T. B., JR. AND MOORE, G. E.—(1949) *J. nat. Cancer Inst.*, **10**, 303.
KLEIN, E., KURNICK, N. B. AND KLEIN, G.—(1950) *Exp. Cell. Res*, **1**, 127.
KREIG, A.—(1953) *Klin. Wschr.*, **31**, 350.
MELLORS, R. C., GLASSMAN, A. AND PAPANICOLAOU, G. N.—(1952) *Cancer*, **5**, 458.
METCALF, R. L. AND PATTON, R. L.—(1944) *Stain Tech.*, **19**, 11.
MOBERGER, G.—(1954) *Acta radiol.* Suppl., **112**, 1.
PEACOCKE, A. R. AND SKERRETT, J. N. H.—(1956) *Trans. Faraday Soc.*, **52**, 261.
PRICE, J. M. AND LAIRD, A. K.—(1950) *Cancer Res.*, **10**, 650.
SIMON, T. R. AND RICCI, A.—(1957) *Trans. Intersoc. Cytology Coun.*, **5**, 121.
VINEGAR, R.—(1956) *Cancer Res.*, **16**, 900.